# BMSC-Derived Exosomal miR-21a-5p Ameliorates Blood–Brain Barrier Injury and Hemorrhagic Transformation

**DOI:** 10.1007/s12035-025-05650-6

**Published:** 2026-01-08

**Authors:** Jing Yang, Ning Tang, Ruanxian Dai, Jiajie Chen, Zhaojiao Li, Fengwen Jiang, Shiyi Li, Qiang Meng

**Affiliations:** 1 Faculty of Life Science and Technology, Kunming University of Science and Technology, Kunming, 650500 P.R. China; 2 Department of Neurology, the Affiliated Hospital of Kunming University of Science and Technology, the First People’s Hospital of Yunnan Province, Kunming, 650032 Yunnan China; 3 Medical School, Kunming University of Science and Technology, Kunming, 650500 P.R. China; 4 College of Science, Minzu University of China, Beijing, 100081 China

**Keywords:** Stroke, Hemorrhagic transformation, Blood–brain barrier, Bone marrow stromal cell, Exosome

## Abstract

Hemorrhagic transformation (HT) and disruption of the blood–brain barrier (BBB) injury are critical pathological features of ischemic stroke, profoundly influencing the natural course of the disease and treatment outcome. This study aims to elucidate how bone marrow stromal cell-derived exosome (BMSC-exo) influence BBB injury and HT and the related molecular mechanisms, focusing on the core function of exosome miR-21a-5p. The HT model induced by middle cerebral artery occlusion (MCAO-HT) was treated with tail vein injection of BMSCs-exo, and HT, BBB injury and brain damage were assessed by mNSS score, triphenyltetrazolium chloride (TTC) staining, evan’s blue (EB)/hemoglobin leakage assay, and HE staining. High abundance miRNAs in BMSC-exo were identified by Small RNA sequence. BMSC-exo miR-21a-5p was modulated using inhibitor and mimic, following by exploring the function of miR-21a-5p in an MCAO-HT animal model and an BBB model. BMSCs-exo effectively enhanced neurological recovery, minimized infarct size, mitigated BBB leakage, and upregulated the expression of Claudin-5/Occludin/ZO-1 in the MCAO-HT model. Five hundred and seventy-one miRNAs were identified in BMSCs-exo, and the targets of the TOP 10 miRNAs in abundance were enriched in TLR, NF-κB, and MMPs pathways. In in vitro experiments, BMSCs-exo rescued oxygen glucose deprivation/reperfusion-induced BBB injury by delivering miR-21a-5p, characterized by an increase in trans epithellal electric resistance and tight junction protein levels and a decrease in permeability, which was dependent on the inhibition of TLR2/NF-κB pathway and the levels and enzymatic activities of MMP2/9. In in vivo experiments, miR-21a-5p knockdown impaired the neuroprotective effects of BMSCs-exo, whereas miR-21a-5p overexpressing lentivirus alone significantly ameliorated brain injury and inhibited the TLR2/NF-κB pathway and the levels and enzymatic activities of MMP2/9 in the MCAO-HT model. BMSCs-exo mitigates BBB disruption and HT by delivering miR-21a-5p to inhibit the TLR2/NF-κB pathway, downregulate the levels and enzymatic activities of MMP-2/9 and protect tight junction proteins.

**Supplementary Information:**

The online version contains supplementary material available at 10.1007/s12035-025-05650-6.

## Introduction

Hemorrhagic transformation (HT), a severe complication of ischemic stroke (IS), occurs when ischemic brain tissue evolves into hemorrhagic lesions following reperfusion therapy or spontaneous reperfusion [1, 2]. The pathophysiological cascade leading to HT is inextricably linked to the severe injury of the blood–brain barrier (BBB) [1–3]. Following ischemia and subsequent reperfusion (I/R), the highly specialized cerebrovascular endothelium, cemented by tight junction proteins, suffers a catastrophic loss of integrity. This breach is driven by a multifaceted process involving intense inflammatory responses, oxidative stress, and crucially, the upregulation of matrix metalloproteinases (MMPs), especially MMP-9 MMP-2, which injure the basal lamina and tight junction components [1–5]. The consequent failure of the BBB results in increased permeability of vascular, accelerating the extravasation of erythrocytes and hemoglobin into the brain parenchyma—the hallmark of HT [1–5]. This hemoglobin release then acts as a potent toxin, fueling further oxidative damage and inflammation, thereby establishing a vicious cycle that expands the core injury [1–5]. Therefore, the imperative to develop strategies that directly target and preserve BBB integrity is paramount for mitigating HT and improving the prognosis of IS.

Bone marrow mesenchymal stem cells (BMSCs) can effectively ameliorate cerebral ischemic injury and neurological function due to their immunomodulatory function, paracrine effect, and multidirectional differentiation potential [6, 7]. Nevertheless, BMSC grafts still faces challenges such as low homing efficiency and survival, complex preparation and preservation processes, and potential tumorigenicity [8]. Notably, the neuroprotective and reparative functions of BMSCs are attributed in part to their secreted extracellular vesicles, especially exosomes [8, 9]. BMSC-derived exosomes (BMSC-exo) have the advantages of high biosafety, easy storage and transportation, low immunogenicity, and penetration of biological barriers [10, 11]. In particular, the penetration of biological barrier provides more possibilities for the repair of BBB damage. Accumulating studies have demonstrated that BMSC-exo effectively mitigates the I/R process in in vivo and in vitro models by modulating autophagy [12], pyroptosis [13], oxidative stress and inflammation [14], synapses and neural plasticity [15, 16], and angiogenesis [17, 18]. However, the function of BMSC-exo in BBB damage and HT repair is unknown. Notably, most studies have confirmed that miRNAs are the molecular mechanisms of BMSC-exo exerting neuroprotective and reparative functions, including miR-455-3p [19], miR-148b-3p [20], miR-133a-3p [12], miR-193b-5p [21], miR-15a-5p [22], miR-206 [14], miR-486 [23], and miR-21-5p [18], suggesting a critical role for miRNAs.

In summary, this study intends to elucidate how BMSC-exo influence BBB injury and HT by constructing in vivo and in vitro models. Moreover, miRNA profiles in BMSC-Exo were systematically characterized using Small RNA sequencing to screen for key candidate miRNAs. Finally, the core functions of the candidate miRNAs in mediating the protective effects of BMSC-exo was explored by rescue experimental strategies in in vivo and in vitro models. This study seeks to establish a critical experimental foundation for comprehensively elucidating the neurovascular protective mechanisms of BMSC-Exo, thereby laying a robust theoretical groundwork for advancing cell-free therapeutic approaches utilizing exosomes or their principal effector molecules, such as miRNAs.

## Materials & Methods

### BMSCs and Exosomes

For BMSC isolation, C57BL/6 J mice were euthanized using cervical dislocation after CO₂ asphyxia, followed by isolation of the femur and tibia. Heparin-containing PBS was used to rinse the marrow cavity to collect bone marrow cell suspensions. After preparation of single cell suspensions, cells were inoculated in T25 bottles containing 15% specialty FBS (12,664,025; Gibco, USA), 1% P/S (15,140,163; Gibco), and α-MEM (12,571,048; Gibco) at a density of 1 × 10^6^ cells/cm^2^. Fresh complete medium was changed every 2–3 days to remove unattached hematopoietic cells and cellular debris. When cells in primary culture grew to approximately 70–90% fusion, digestion and passaging were performed using 0.25% trypsin–EDTA solution (25,200,056; Gibco). P3 BMSC were taken for identification of surface markers using flow cytometry. Flow cytometry antibodies were: rat anti-CD34 mAb (14–0341-82; Invitrogen, USA; RRID: AB_467210), rat anti-CD45 mAb with FITC (11–0451-82; Invitrogen; RRID: AB_465050), rat anti-CD44 mAb (14–0441-82; Invitrogen; RRID: AB_467246), and mouse anti-CD45 mAb with PE (12–0900-83; Invitrogen; RRID: AB_465774).

For BMSC-exo isolation, the supernatant of BMSCs was successively centrifuged at 300 × g, 2000 × g and 10,000 × g for 10 min, 10 min and 30 min, respectively, to eliminate intact cells, dead cells and cellular debris. Subsequently, Ribo Exosome Isolation Reagent (C10130; Ribobio, China) was added to the supernatant after centrifugation and incubated overnight. Then, the mixture underwent a 30-min centrifugation at 15,000 × g to yield the exosome precipitate. The markers and morphological characteristics of BMSC-exo were identified by western blotting and transmission electron microscopy (TEM). Antibodies included: rat anti-ALIX mAb (ab186429; Abcam, USA; RRID: AB_2754981), rabbit anti-TSG101 mAb (ab125011; Abcam; RRID: AB_10974262), and rabbit anti-CD9 pAb (ab223052; Abcam; RRID: AB_2922392). Further, BMSC-exo was quantitatively analyzed using the ExoQuick-TC Kit (EXOCET96A-1; SBI, USA) and a microplate reader (Multiskan SkyHigh; Thermo Scientific, USA) at a wavelength of 405 nm. Based on the standard curve (y = 0.02774x + 0.01266), the concentration range of BMSC-exo isolated in this study batch was 8.124534 × 10^8^ – 9.822787 × 10^8^ exosomes/µL. The concentration of BMSC-exo was adjusted to 10^8^ exosomes/µL for subsequent experiments.

### Cell Transfection and BBB Model

miR-21a-5p inhibitor and mimic were constructed by Ribobio (China). BMSCs were transfected with inhibitor (100 nM) and mimic (50 nM) using riboFECT CP (C10511-05, Ribobio) to knock down and overexpress miR-21a-5p in exosomes. After 48 h of transfection, BMSCs and exosomes were taken to characterize their efficiency using qRT-PCR. The effects of BMSC-exo and miR-21a-5p on BBB injury were assessed in vitro using the BBB model. As mentioned earlier [24], a BBB model was constructed using astrocytes and mouse brain microvascular endothelial cells (BMECs), and HT and BBB injury in vitro was produced through oxygen–glucose deprivation/reoxygenation (OGD/R). The BBB model was randomized into: the BBB, OGD/R, BMSC-exo, Exo^Inhibitor miR−21a−5p^, and Exo^mimic miR−21a−5p^ groups (Fig. 5C). Except for the BBB group, the BBB model underwent OGD/R to induce injury. OGD/R was induced by hypoxia (1% O_2_, 5% CO_2_, and 94% N_2_,) and glucose-free DMEM medium for 2 h. Subsequently, BBB model was induced by normoxia (21% O_2_ and 5% CO_2_) and standard DMEM medium for 24 h. Before and after the OGD/R procedure, the BBB models in the BMSC-exo, Exo^Inhibitor miR−21a−5p^, and Exo^mimic miR−21a−5p^ groups were treated with standard BMSC-exo, BMSC-exo transfected with miR-21a-5p inhibitor, and BMSC-exo transfected with miR-21a-5p mimic, respectively.

### Animals and MCAO-HT Model

C57BL/6 J mice (male; 18–22 g), sourced from the Kunming Medical University, were maintained under SPF conditions with a 12-h light–dark cycle, strictly controlled ambient temperature of (23 ± 1) °C, and relative humidity maintained between (55–65) %. All animal procedures were approved by the Kunming Medical University (kmmu20241922), and the ARRIVE guidelines were strictly adhered to in order to minimize pain and number of mice.

Mice were randomized into: the Sham (*N* = 15), MCAO-HT (*N* = 30), BMSC-exo (*N* = 30), Exo^Inhibitor miR−21a−5p^ (*N* = 15), and LV-mimic miR-21a-5p (*N* = 15) groups. The mouse HT model induced by middle cerebral artery occlusion (MCAO-HT), mNSS scoring, TTC staining, evan’s blue (EB) leakage assay, and hemoglobin content assay were performed as we previously described ^24–26^. For the MCAO-HT procedure, mice were anesthetized with 0.3% pentobarbital and the right common carotid artery (CCA), internal carotid artery (ICA), and external carotid artery (ECA) were exposed. A hemostatic clamp was then placed to occlude the distal end of the CCA. Two 6–0 sutures were used to tie a knot at the distal end of the ECA and a slipknot at the intersection of the CCA and the ECA. An incision was created between the two knots, followed by insertion of the silicone thread plug. After the silicone thread plug reaches the MCA (the marked point on the thread plug), the slipknot at the intersection of the CCA and ECA was tightened. After 5 h of continuous anesthesia with isoflurane gas, the silicone thread plug was removed, and the neck skin was sutured. Sham-operated mice were exposed only to the CCA, ICA, and ECA. After the MCAO-HT procedure, mice in the BMSC-exo, Exo^Inhibitor miR−21a−5p^, and LV-mimic miR-21a-5p groups were injected in the tail vein with 100 µL of BMSC-exo, 100 µL of BMSC-exo transfected with miR-21a-5p inhibitor and 100 µL of miR-21a-5p mimic lentivirus (5 × 10^7^ TU). One week after treatment, mice were euthanized using cervical dislocation after CO₂ asphyxia. The entire brain was used for histological staining, while brain tissue on the side of ischemic injury was used for molecular experiments. Brain tissue sections stained with TTC were subjected to HT scoring according to the following criteria [24, 27]: 0 indicates no hemorrhage; 1 indicates small punctate hemorrhages at the infarct margin; 2 indicates punctate hemorrhages within the infarct area; 3 indicates hemorrhage area less than 30% of the infarct area; 4 indicates hemorrhage area greater than or equal to 30% of the infarct area.

### Histological Staining

The effects of BMSC-exo and miR-21a-5p on pathological damage of mouse brain tissue was observed using HE and TUNEL staining. Fluorescence intensity and localization of Claudin-5 and ZO-1 in mouse brain tissue was observed using immunofluorescence staining. Immunofluorescent antibodies were: rabbit anti-Claudin-5 pAb (PA5-99,415; Invitrogen; RRID: AB_2818348), rabbit anti-ZO-1 pAb (ab216880; Abcam; RRID: AB_2909434), and goat anti-rabbit IgG (H + L) pAb (A-11008; Invitrogen; RRID: AB_143165). The specific methods of histological staining are as we previously described [24].

### Small RNA Sequence and Bioinformatics

miRNAs in BMSC-exo were identified using small RNA sequence, and bioinformatics analysis was used to characterize the abundance of miRNAs and the biological functions and potential molecular mechanisms of their targets. The small RNA sequence was performed by Oebiotech (China). The detailed methods for small RNA sequence and Bioinformatics are as we previously described [24].

### qRT-PCR Assay

The levels of top10 miRNAs with high abundance were detected in mouse brain tissues on the side of ischemic injury, BMSCs, BMSC-exo, and BMECs using qRT-PCR. The detailed methodology of the qRT-PCR assay is as we previously described [24]. The expression of the target gene relative to the internal reference (U6) was calculated.

### Western Blotting Assay

The expression of Claudin-5, MMP-2, TLR2, Occludin, MMP-9, p-P65, ZO-1, and P65 in mouse brain tissues on the side of ischemic injury and BMECs was detected using western blotting assay. Western blotting antibodies were: rabbit anti-Claudin-5 pAb (PA5-99,415; RRID: AB_2818348; Invitrogen), rabbit anti-ZO-1 pAb (ab216880; RRID: AB_2909434; Abcam), rabbit anti-MMP-2 pAb (bs-0412R; RRID: AB_10856777; Bioss, China), rabbit anti-Occludin mAb (ab216327; RRID: AB_2737295; Abcam), rabbit recombinant anti-MMP- 9 (ab283575; RRID: AB_2928971; Abcam), rabbit anti-TLR2 mAb (ab209216; RRID: AB_3662636; Abcam), rabbit recombinant anti-p-P65 (Ser468) (82,335–1-RR; RRID: AB_3083091; Proteintech, USA), rabbit recombinant anti-P65 (80,979–1-RR; RRID: AB_2918923; Proteintech) and mouse anti-β-actin mAb (ab8226; RRID: AB_306371; Abcam). The detailed methodology of western blotting assay is as we previously described [24]. The gray values of each gel blot were analyzed using Image Lab software, and the expression of the target protein relative to the internal reference (β-actin) was calculated.

### ELISA Assay

The levels of MMP-2 and MMP-9 in the brain tissue homogenate of the ischemic injury side and the supernatant of the BBB model were detected using MMP-2 (E-EL-M0780; Elabscience, China) and MMP-9 (E-EL-M3052; Elabscience, China) ELISA Kits. After centrifugation of the brain tissue homogenate and culture supernatant, 100 µL of the supernatant was added to the sample wells of the ELISA plate. Meanwhile, the same volume of the standard solution and sample diluent was added to the standard wells and blank wells respectively. The samples in each well were sequentially treated with 100 μL biotinylated antibod, 350 μL wash buffer, 100 μL HRP enzyme conjugate solution, 90 μL TMB solution, and 50 μL stop solution for 60 min, 1 min, 30 min, and 15 min, respectively. Finally, the samples in ELISA plate were quantitatively analyzed using a microplate reader (Multiskan SkyHig) at a wavelength of 450 nm, and the concentrations of MMP-2 and MMP-9 were calculated based on the standard curve.

### Detection of MMP2 and MMP9 Enzyme Activities

The enzymatic activities of MMP2 and MMP9 in the ischemic brain tissue and BMECs of mice were determined using the MMP Activity Assay Kit (Fluorometric–Green) (ab112146, Abcam). The protein samples from brain tissue and BMECs were measured for concentration at 562 nm using the Enhanced BCA Protein Assay Kit (BL1054S; Biosharp, China). Equal volumes of 2 mM 4-Aminophenylmercuric Acetate were added to 96-well plates containing protein samples and incubated at 37 °C for 1 h and 2 h, respectively, to activate MMP2 and MMP9. The total volume of each well was 50 μL. Subsequently, 50 μL of diluted MMP Green Substrate Working Solution was added to each well of the 96-well plate. After incubation for 60 min, the fluorescence of each well was detected at an excitation/emission wavelength of 490/525 nm using a BioTek Synergy H1 microplate reader (Agilent, USA). Subsequently, the enzymatic activities of MMP2 and MMP9 were calculated and evaluated as RFU/μg protein.

### Statistical Analysis

Data distribution normality was verified through application of the Shapiro–Wilk test. Data conforming to a normal distribution were displayed as mean ± standard deviation (SD) and analyzed by one-way analysis of variance. Data not conforming to a normal distribution were presented as interquartile ranges and analyzed using the Kruskal–Wallis test. Results were deemed statistically significant at* P* < 0.05*.* Statistical analyses and corresponding visualizations were performed through GraphPad Prism software.

## Results

### BMSC-Exo Ameliorates BBB Injury and HT in a MCAO-HT Animal Model

**Fig. 1:**
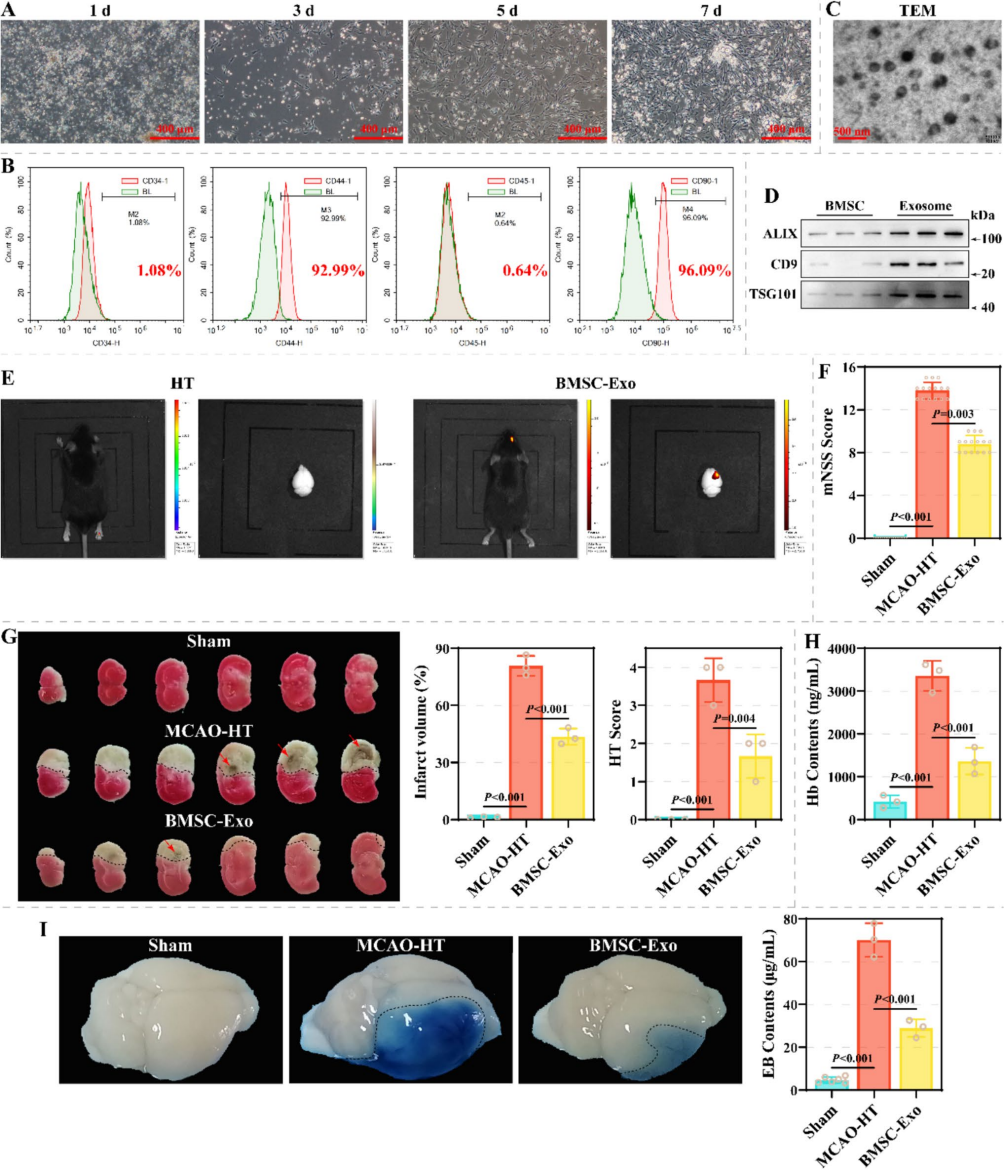
Effects of BMSC-exo on BBB injury and HT in a MCAO-HT animal model. **A** Representative images of P0 BMSCs at days 1, 3, 5 and 7. Scale: 400 µm. **B** Surface marker profiles (CD34, CD45, CD44, and CD90) of BMSCs were characterized by flow cytometry (*N* = 3). **C** Representative images of BMSCs-exo, characterized by TEM (*n* = 3). Scale: 500 nm. **D** The expression of ALIX, CD9 and TSG101 in BMSCs and BMSCs-exo was assessed by Western blotting assay (*N* = 3). **E** The distribution of BMSCs-exo in the mouse brain was tracked using a small animal in vivo imaging system (*N* = 6). **F** The effect of BMSC-exo on neurological function of MCAO-HT animal models was characterized by mNSS scores (*N* = 15). **G** The effect of BMSC-exo on infarct volume and HT score of MCAO-HT animal models was assessed by TTC staining (*N* = 3). **H**-**I** The effects of BMSC-exo on HT and BBB injury of MCAO-HT animal models were assessed by hemoglobin content (H) and EB leakage assay (**I**) (*N* = 3)

The growth status and morphology of P0 BMSCs on days 1, 3, 5 and 7 are shown in Fig. 1A. P0 BMSCs exhibited adherent growth, characterized by typical fusiform, stellate and polygonal-like shapes, accompanied by colony formation and contact inhibition. Moreover, surface markers were characterized as CD34^−^CD45^−^CD44^+^CD90^+^ (Fig. 1B). TEM revealed that BMSCs-exo presented a double-layered membrane in the form of a cup (diameter: around 120 nm) and the electron densities were not homogeneous (Fig. 1C). Compared to BMSCs, exosomes were enriched in ALIX, CD9 and TSG101 (Fig. 1D). These results are consistent with the characterization of BMSCs and exosomes.

**Fig. 2:**
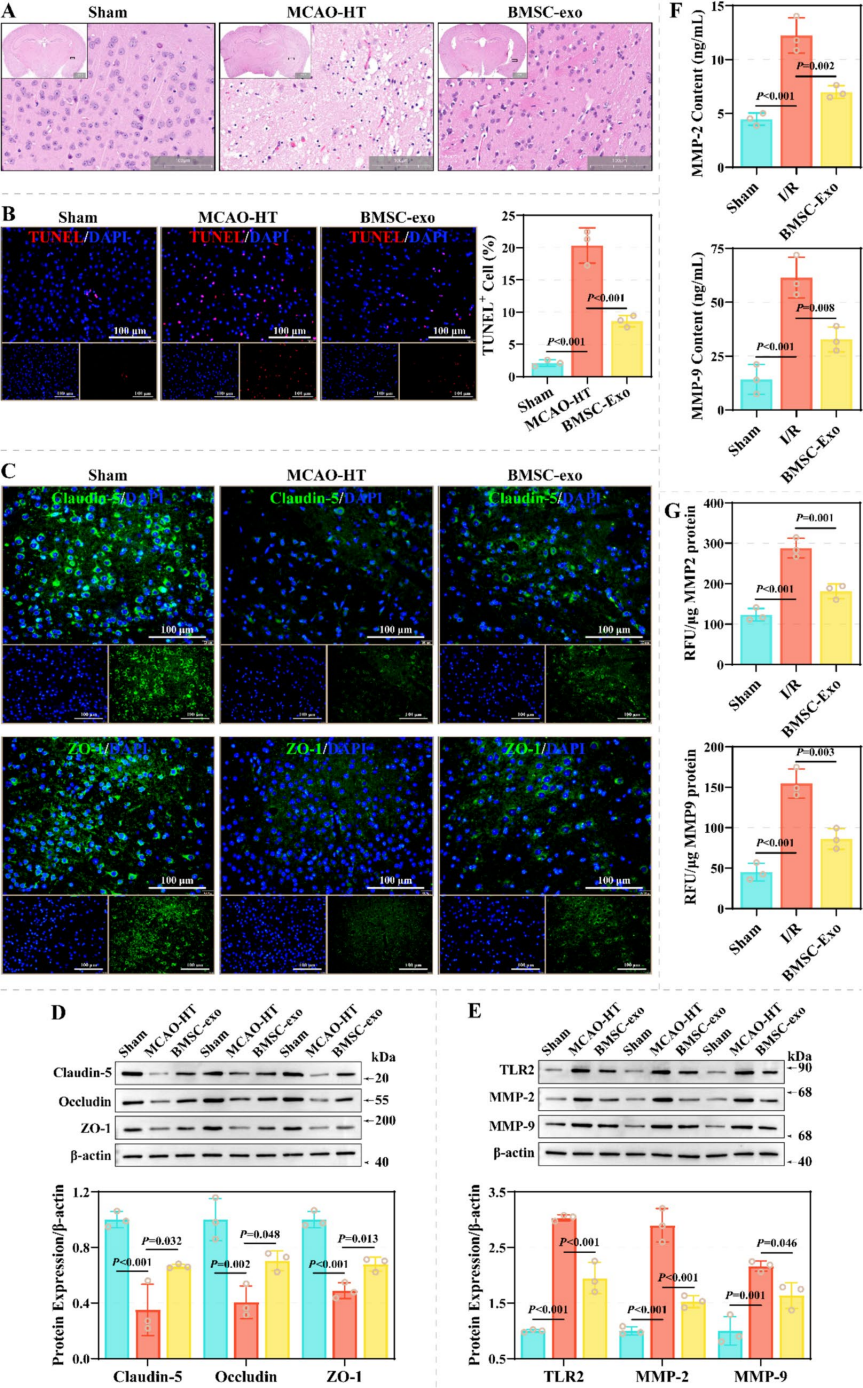
Effect of BMSC-exo on pathological damage and marker expression in a MCAO-HT animal model. **A** Representative images of brain tissue from the Sham, MCAO-HT, and BMSC-Exo groups, characterized by HE staining (*N* = 3). Scale: 100 µm. **B** The effect of BMSC-exo on apoptosis in brain tissue of MCAO-HT model was observed by TUNEL staining (*N* = 3). **C** Representative images of Claudin-5 and ZO-1 in brain tissue on the side of ischemic injury from the Sham, MCAO-HT, and BMSC-Exo groups, characterized by immunofluorescence staining (*N* = 3). Scale: 100 µm. **D**-**E** The effect of BMSC-exo on the levels of Claudin-5, Occludin, and ZO-1 **(D)** and TLR2, MMP-2, and MMP-9 **(E)** in brain tissues on the side of ischemic injury of MCAO-HT animal models (*N* = 3). **F**-**G** Differences in the levels (**F**) and enzymatic activities (**G**) of MMP-2 and MMP-9 in brain tissue homogenates on the side of ischemic injury between the Sham, MCAO-HT, and BMSC-Exo groups (*N* = 3)

Further, the present study injected BMSC-exo via tail vein in a MCAO-HT model and subsequently assessed the integrity of the BBB and HT. In vivo imaging system revealed that tail vein-injected BMSC-exo homed to the mouse brain (Fig. 1E). BMSC-exo resulted in a decrease in mNSS scores from 14 (13, 14) to 9 (8, 9) in the MCAO-HT model (Fig. 1F). TTC staining indicated that BMSC-exo treatment effectively resulted in a decrease in infarct volume and HT scores from (80.80 ± 5.34) % and 4 (3, 4) to (43.62 ± 4.30) % and 2 (1, 2) (Fig. 1G). Additionally, hemoglobin and EB levels in brain tissue on the side of ischemic injury decreased from (3357 ± 347.3) ng/mL and (70.16 ± 7.90) µg/mL to (1366 ± 311.3) ng/mL and (28.92 ± 4.10) µg/mL (Fig. 1H-1I). HE staining revealed that compared to the Sham group, the brain tissue structure was obviously damaged in the MCAO-HT group, characterized by edema, erythrocyte extravasation, neuronal necrosis (ghost cells), formation of foam cells, and infiltration of inflammatory cells (Fig. 2A). TUNEL staining exhibited that the proportion of apoptotic cells in the brain tissue from the MCAO-HT model was observably increased (Fig. 2B). These pathologic features improved after BMSC-exo treatment. Mechanistically, the levels of Claudin-5, Occludin, and ZO-1 were remarkably increased in brain tissue on the side of ischemic injury after BMSC-exo treatment (Fig. 2C-2D). Moreover, BMSC-exo resulted in a decrease in protein levels of TLR2, MMP-2 and MMP-9 in brain tissue on the side of ischemic injury from 3.033 ± 0.055, 2.898 ± 0.303 and 2.165 ± 0.095 to 1.947 ± 0.280, 1.526 ± 0.106 and 1.636 ± 0.230 (Fig. 2E). Similarly, ELISA assay demonstrated that BMSC-exo effectively reduced the levels and enzymatic activities of MMP-2 and MMP-9 in the brain tissue homogenates on the side of ischemic injury form the MCAO-HT model (Fig. 2F-2G). These results suggest that BMSC-exo effectively ameliorated BBB damage and HT in I/R.

### Identification of miRNAs in BMSCs-Exo

**Fig. 3:**
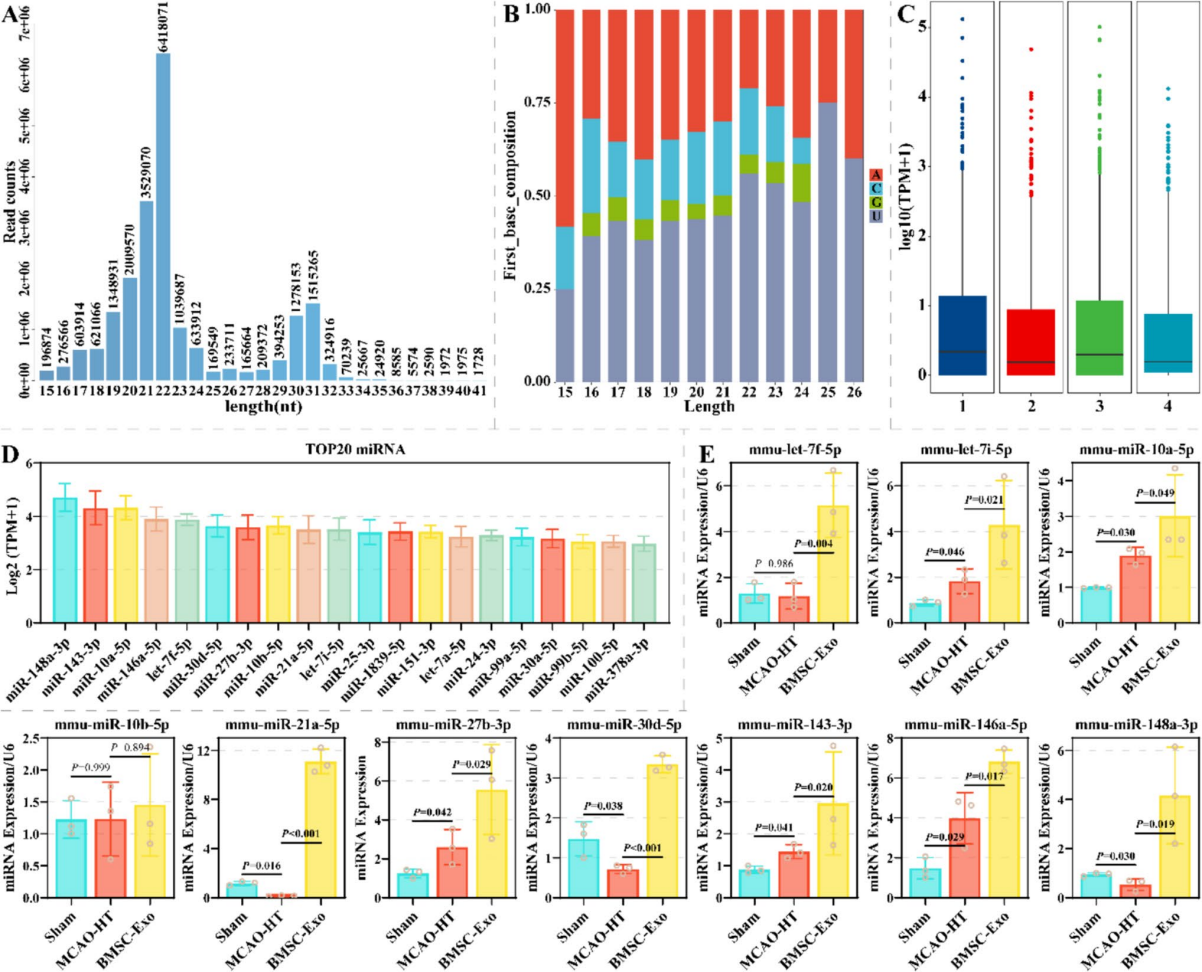
Identification of miRNAs in BMSC-exo. **A** The histogram showing the length distribution of clean reads in BMSC-exo (*N* = 4). **B** Preference of different length miRNAs for the first base in BMSC-exo (*N* = 4). **C** Box plots illustrating the expression and symmetry of miRNAs in four biological replicates (*N* = 4). **D** The top 20 miRNAs with the highest levels in BMSC-exo, characterized by log_2_(TPM + 1) (*N* = 4). **E**: The expression differences of the top 10 miRNAs with the highest levels were detected by qRT-PCR in the brain tissues on the side of ischemic injury from the Sham, MCAO-HT, and BMSC-Exo groups (*N* = 3)

This study identified miRNAs in BMSCs-exo using Small RNA sequence to investigate the potential molecular mechanisms by which BMSCs-exo mediates BBB injury and HT. Clean reads are predominantly distributed in lengths of 17–24 and 29–32 nt, which may be miRNAs and piRNAs/tRNAs/rRNAs (Fig. 3A). After miRNA annotation, base preference statistics revealed that the first base of miRNAs (15–26 nt) favored U and was resistant to G (Fig. 3B). After calibration, the median values of miRNAs expression for the four biological replicates were essentially identical (Fig. 3C). A total of 174 miRNA families and 571 miRNAs were identified in BMSCs-exo (Supplementary File 1). Top 20 miRNAs with high abundance are shown in Fig. 3D. This study verified the top 10 miRNAs with high abundance in brain tissues on the side of ischemic injury of the Sham, MCAO-HT, and BMSC-Exo groups using qRT-PCR. Let-7f-5p, let-7i-5p, miR-10a-5p, miR-21a-5p, miR-27b-3p, miR-30d-5p, miR-143-3p, miR-146a-5p and miR-148a-3p exhibited differential expression across the three groups (Fig. 3E). Notably, miR-21a-5p showed the greatest difference among the Sham, HE, and BMSC-Exo groups. These results suggest that these miRNAs may be related to BMSCs-exo mediating BBB injury and HT.

**Fig. 4:**
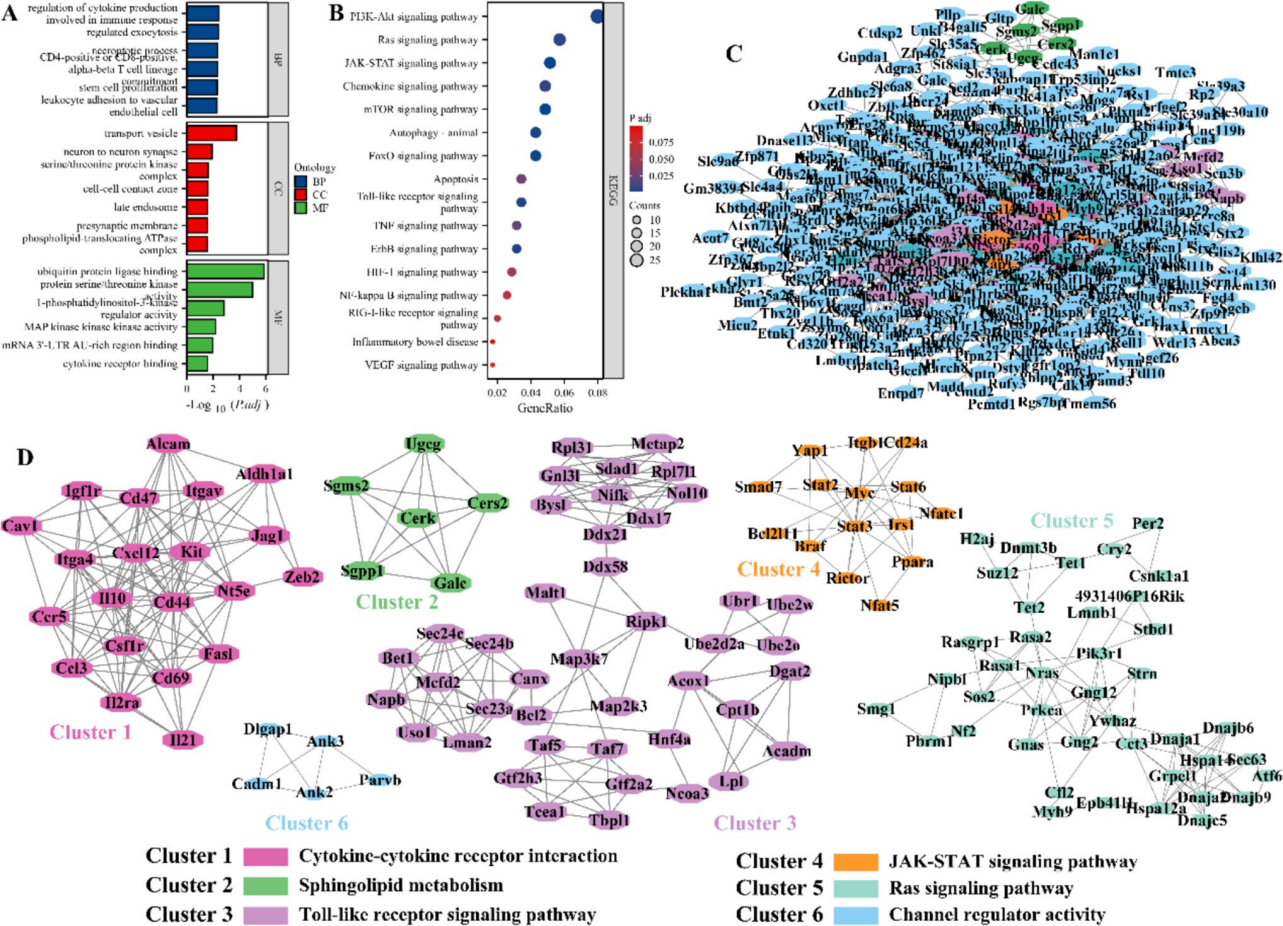
Functions and molecular mechanisms of TOP10 miRNA targets in BMSC-exo. **A**-**B** Bar plot **(A)** and bubble plot **(B)** showing the GO terms and KEGG pathways to which the targets of TOP10 miRNAs are enriched. **C** PPIs constructed from targets of TOP10 miRNAs. **D** Critical sub-networks in PPIs were identified using the MCODE algorithm and the biological functions involved in each sub-network

This study characterized the biological functions and potential molecular mechanisms of the targets of top10 miRNAs with high abundance. GO and KEGG enrichment analyses revealed that these targets mainly regulate neuritogenesis, differentiation and death, immunity, cell cycle, inflammatory response, cytophagy, anoxia, angiogenesis, and glucose metabolism, as well as are associated with PI3K-AKT, Ras, JAK-STAT, mTOR, FoxO, Toll-like receptor, TNF, NF-κb, HIF-1 and VEGF pathways (Fig. 4A-4B and Supplementary File 2). This study further constructed a protein–protein interaction (PPI) network to characterize hub genes in these targets. This PPI network contains 2699 relationship pairs and 667 nodes, of which the TOP 10 genes with connectivity are *Stat3*, *Bcl2*, *Myc*, *Ubc*, *Itgb1*, *Cd44*, *Mapk14*, *Il10*, *Braf*, and *Skp1a* (Fig. 4C and Supplementary File 3). Since the network was too large for screening key genes, the MCODE algorithm was used in this study for clustering the sub-network. This PPI network was clustered into six sub-networks, which respectively regulate cytokine-cytokine receptor interaction, sphingolipid metabolism, TLR pathway, JAK-STAT pathway, Ras signaling pathway and channel regulator activity (Fig. 4D). These results suggest that BMSCs-exo miRNAs may mediate a variety of biological behaviors through target-associated multidimensional regulatory networks, thereby affecting BBB damage and HT.

### miR-21a-5p in BMSCs-Exo Improves the Integrity of the BBB Model

**Fig. 5:**
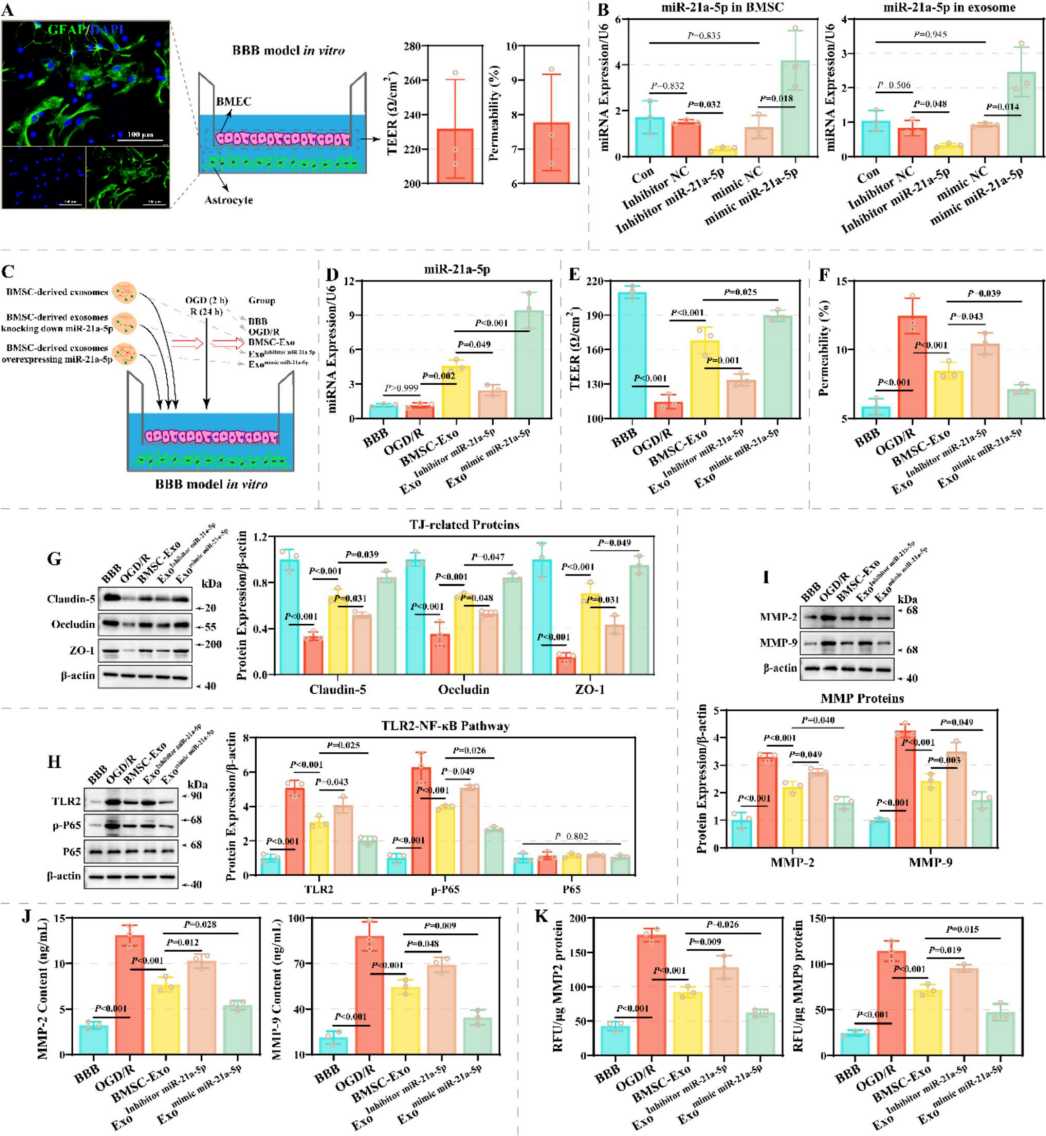
Effect of BMSC-exo on BBB model injury. **A** Schematic diagram of the BBB model and its TEER and permeability characteristics. Scale: 100 µm. **B** qRT-PCR assay was performed to assess the effect of inhibitor and mimic on miR-21a-5p expression in BMSCs and exosomes (*N* = 3). **C** Flowchart and grouping information for the cell experiment. **D** The effects of inhibitor and mimic on miR-21a-5p expression in the BBB model were assessed by qRT-PCR assay (*N* = 3). **E**–**F** The effect of miR-21a-5p inhibitor and mimic on TEER (**E**) and permeability (**F**) in the BBB model (*N* = 3). **G**-**I**: Representative images of Claudin-5, Occludin, and ZO-1 (**G**), TLR2, p-P65, and P65 (**H**), as well as MMP-2 and MMP-9 (**I**), characterized by western blotting assay (*N* = 3). **J**-**K**: Differences in the levels (**J**) and enzymatic activities (**K**) of MMP-2 and MMP-9 in cell supernatant between the BBB, OGD/R, BMSC-Exo, Exo^Inhibitor miR−21a−5p^, and Exo.^Inhibitor miR−21a−5p^ groups (*N* = 3)

Since miR-21a-5p has the most pronounced differential expression in brain tissue, it was selected for subsequent functional experiments. A BBB model consisting of astrocytes and BMEC was constructed in vitro (Fig. 5A). The model has a trans epithellal electric resistance (TEER) greater than 200 Ω/cm^2^ and a permeability of less than 10%, suggesting that the model is successful. Moreover, this study transfected inhibitor and mimic in BMSCs for expression regulation of miR-21a-5p in BMSCs-exo. qRT-PCR results revealed that inhibitor effectively reduced miR-21a-5p expression in BMSCs and exosomes, while mimic had the opposite effect (Fig. 5B). Further, the BBB model was treated using BMSCs-exo transfected with inhibitor and mimic, and OGD/R was performed (Fig. 5C). Notably, OGD/R did not alter miR-21a-5p levels in BMEC (Fig. 5D). Moreover, BMSCs-exo treatment remarkably upregulated miR-21a-5p levels in BMEC, which was attenuated by an inhibitor and potentiated by a mimic. OGD/R resulted in reduced TEER and increased permeability, which was rescued by BMSCs-exo (Fig. 5E-5F). miR-21a-5p inhibitor limited the therapeutic effect of BMSCs-exo, whereas miR-21a-5p mimic showed the opposite modulation pattern. Among the mechanism aspects, compared to the BBB group, the levels of Claudin-5, Occludin and ZO-1 were remarkably decreased and the levels of TLR2, p-P65, MMP-2, and MMP-9 were remarkably increased in the OGD/R group (Fig. 5G-5I). Similarly, ELISA assay demonstrated that miR-21a-5p knockdown limited the inhibition of the levels and enzymatic activities of MMP-2 and MMP-9 by BMSC-exo, while miR-21a-5p overexpression exhibited an opposite regulatory pattern (Fig. 5J-5K). Notably, BMSCs-exo alleviated OGD/R-induced changes in these proteins, which were restricted by inhibitor and enhanced by mimic. These results suggest that BMSCs-exo miR-21a-5p alleviated injury in the BBB model in vitro, which is associated with the TLR2/NF-κB pathway.

### miR-21a-5p in BMSCs-Exo Mitigates Brain Injury in a MCAO-HT Models in vivo

**Fig. 6:**
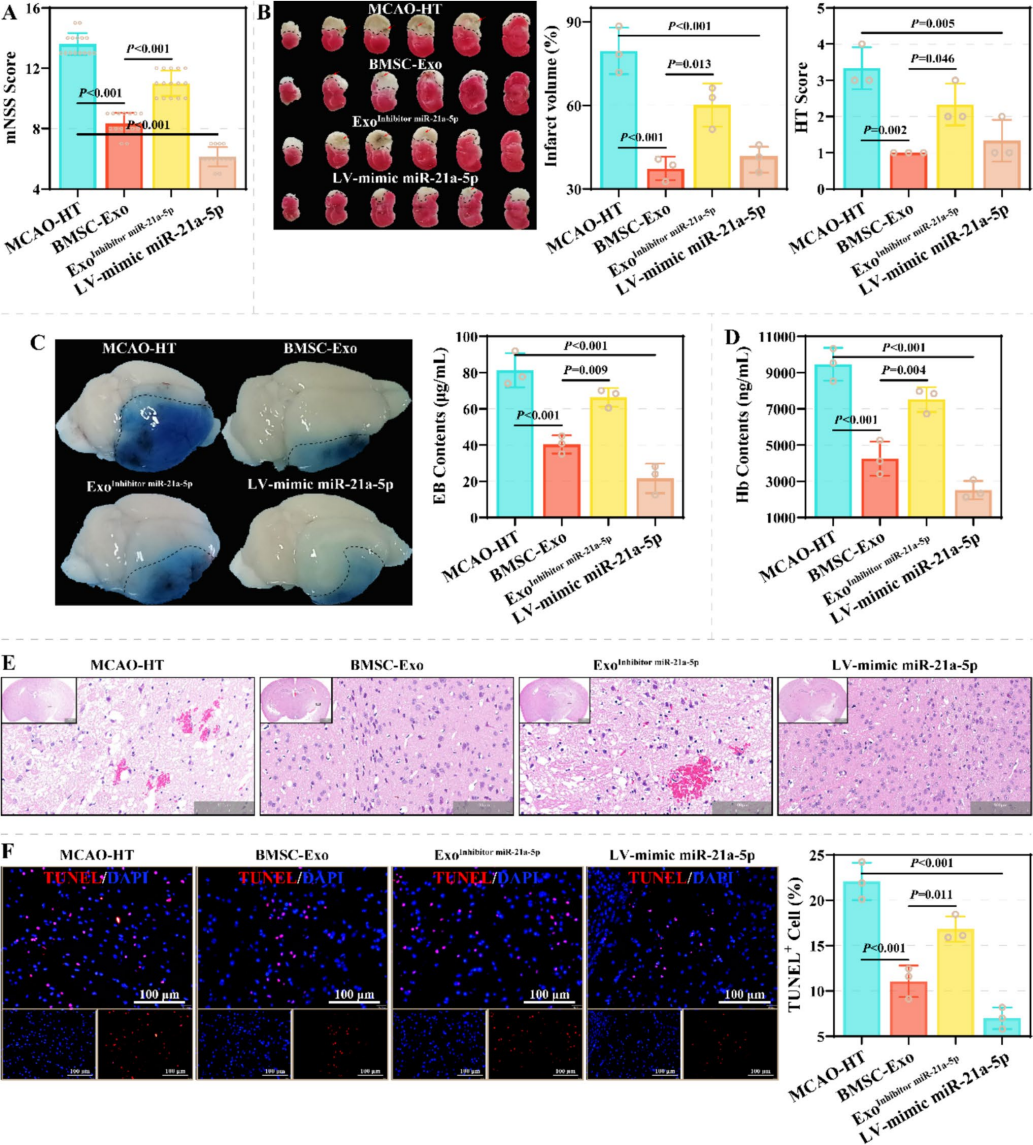
Effect of miR-21a-5p on BBB injury and HT in a MCAO-HT animal model. **A** The effect of miR-21a-5p on neurological function in the MCAO-HT animal model was assessed using the mNSS score (*N* = 15). **B**-**C** Representative images of brain tissue from the MCAO-HT, BMSCs-Exo, Exo^Inhibitor miR−21a−5p^, and LV-mimic miR-21a-5p groups, characterized by TTC staining **(B)** and EB penetration assay **(C)** (*N* = 3). **D** The effect of miR-21a-5p on HT was assessed by hemoglobin content (*N* = 3). **E** The effect of miR-21a-5p on histopathological damage in mouse brain tissue was assessed by HE staining (*N* = 3). Scale: 100 µm.** F**: Representative images of TUNEL staining in brain tissue from the MCAO-HT, BMSC-Exo, Exo.^Inhibitor miR−21a−5p^, and LV-mimic miR-21a-5p groups (*N* = 3)

**Fig. 7:**
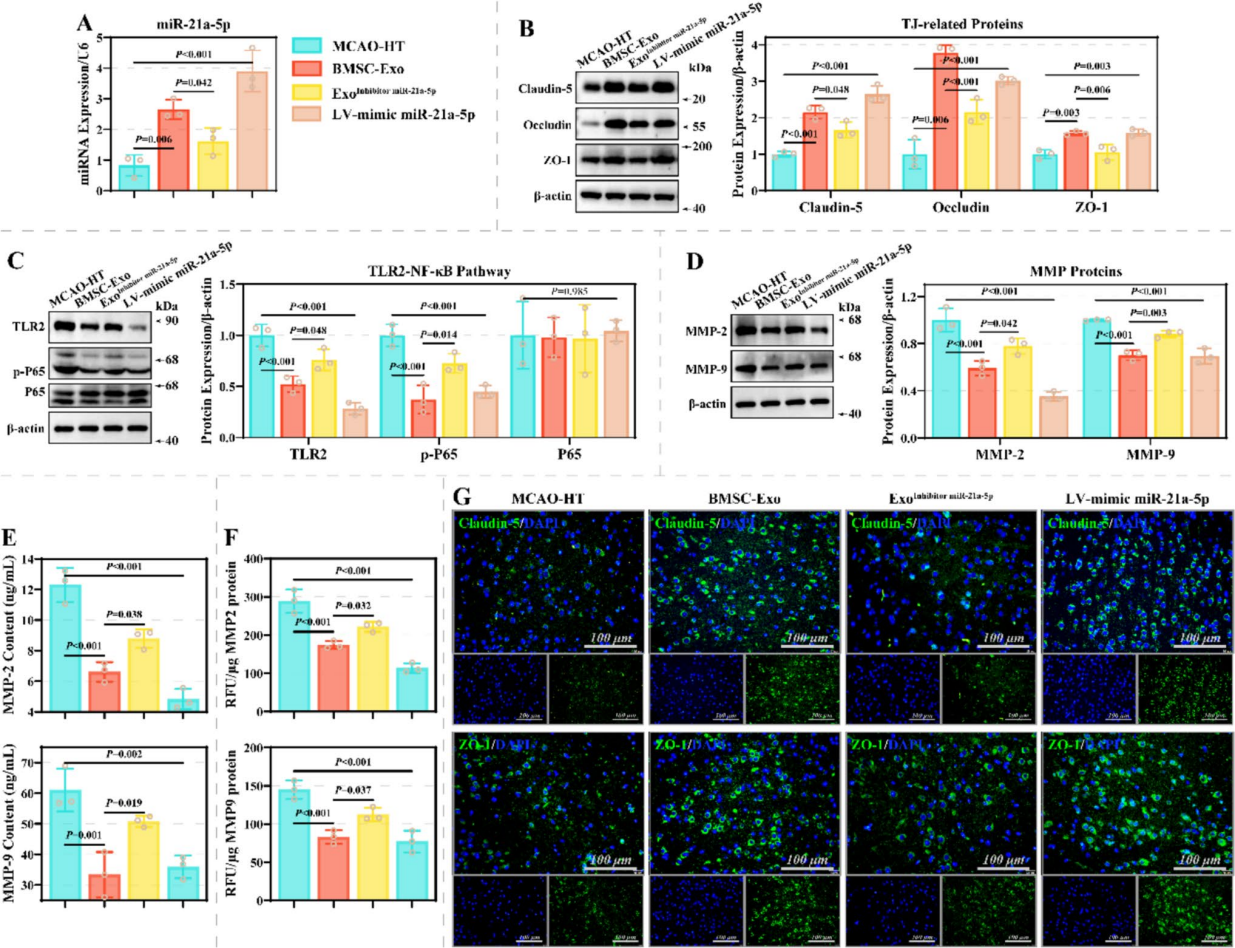
Effect of miR-21a-5p on marker expression in a MCAO-HT animal model. **A**-**D** qRT-PCR and western blotting assays were performed to detect the effects of miR-21a-5p on the expression of miR-21a-5p **(A)**, Claudin-5, Occludin, and ZO-1 **(B)**, TLR2, p-P65, and P65 **(C),** as well as MMP-2 and MMP-9 **(D)** in mouse brain tissues on the side of ischemic injury (*N* = 3). **E**–**F** Differences in the levels (E) and enzymatic activities (**F**) of MMP-2 and MMP-9 in brain tissue homogenates between the MCAO-HT, BMSC-Exo, Exo^Inhibitor miR−21a−5p^, and LV-mimic miR-21a-5p groups (*N* = 3). **G** Representative images of Claudin-5 and ZO-1 in brain tissues on the side of ischemic injury from the MCAO-HT, BMSCs-Exo, Exo^Inhibitor miR−21a−5p^, and LV-mimic miR-21a-5p groups, characterized by immunofluorescence staining (*N* = 3). Scale: 100 µm

Further, this study explored the effects of BMSCs-exo miR-21a-5p on BBB injury and HT in vivo. In this study, BMSCs-exo (BMSCs-Exo group), BMSCs-exo transfected with miR-21a-5p inhibitor (Exo^Inhibitor miR−21a−5p^ group), and miR-21a-5p mimic lentivirus (LV-mimic miR-21a-5p group) were injected via the tail vein to treat the MCAO-HT model. As previously found, BMSCs-exo effectively ameliorated BBB injury and HT in the MCAO-HT model (Fig. 6–7). Compared to the BMSCs-Exo group, mNSS scores of mice in the Exo^Inhibitor miR−21a−5p^ group were markedly increased; compared to the MCAO-HT group, scores in the LV-mimic miR-21a-5p group were markedly decreased (Fig. 6A). miR-21a-5p knockdown limited the inhibitory effects of BMSCs-exo on infarct volume and HT scores, and miR-21a-5p mimic lentivirus effectively improved these features (Fig. 6B). Moreover, miR-21a-5p mimic lentivirus effectively reduced EB and hemoglobin levels in brains, and miR-21a-5p knockdown markedly limited the efficacy of BMSCs-Exo (Fig. 6C-6D). Histopathologically, compared to the BMSC-Exo group, brain tissues in the Exo^Inhibitor miR−21a−5p^ group displayed obvious edema, erythrocyte extravasation, neuronal necrosis, and inflammatory cell infiltration; these features were improved in the LV-mimic miR-21a-5p group in comparison to the MCAO-HT group (Fig. 6E). TUNEL staining further confirmed that miR-21a-5p knockdown limited the inhibitory effect of BMSC-exo on cell apoptosis in brain tissue, while the miR-21a-5p overexpression lentivirus effectively reduced the proportion of TUNEL^+^ cells (Fig. 6F). Molecularly, the levels of miR-21a-5p, Claudin-5, Occludin and ZO-1 were remarkably lower in brain tissues on the side of ischemic injury of the Exo^Inhibitor miR−21a−5p^ group than those of the BMSC-Exo group, whereas TLR2, p-P65, MMP-2, and MMP-9 showed opposite expression patterns (Fig. 7A-7E and Fig. 7G). Additionally, the levels of miR-21a-5p, Claudin-5, Occludin, and ZO-1 were remarkably higher in the LV-mimic miR-21a-5p group than in the MCAO-HT group, whereas TLR2, p-P65, MMP-2, and MMP-9 displayed the opposite expression patterns. The changes in the enzymatic activities of MMP-2 and MMP-9 were consistent with their levels (Fig. 7F). These results suggest that miR-21a-5p contributes to the repair of brain damage by BMSCs-Exo.

## Discussion

Previous studies have emphasized the functions of BMSC-exo in autophagy, pyroptosis, oxidative stress and inflammation, synaptic and neural plasticity, and angiogenesis following I/R [12–18, 28]. This study focused on the role of BMSC-exo on BBB injury and HT and demonstrated that BMSC-exo alleviated the leakage of EB in brain tissue on the side of ischemic injury and enhanced the expression of Claudin-5, Occludin and ZO-1. Additionally, BMSC-exo suppressed HT scores and hemoglobin, as well as the levels and enzymatic activities of MMP-2 and MMP-9. It is well known that Claudin-5, Occludin and ZO-1 are critical proteins that constitute the core structure of the BBB—tight junctions. These structures constitute a tight barrier among cerebral microvascular endothelial cells, precisely controlling the passage of substances from the bloodstream into the brain, and serve as a fundamental component for preserving the homeostasis of the central nervous system microenvironment [29, 30]. Moreover, MMP-9 and MMP-2 are the main performers in the destruction of the BBB, leading to vascular leakage and erythrocyte extravasation, thereby leading to hemoglobin production. Hemoglobin acts as a core toxic mediator after HT, driving intense oxidative stress and inflammation and critically upregulating MMPs, which create a vicious cycle of expanding core pathology [4, 31, 32]. Therefore, the regulatory effects of BMSC-exo on these molecules emphasize that BMSC-exo effectively mitigates BBB damage and HT.

Importantly, this study revealed miRNAs with high abundance in BMSC-exo using small RNA sequences, mainly including miR-21a-5p, miR-148a-3p, miR-24-3p, miR-25-3p, let-7i-5p, miR −10b-5p, miR-30d-5p, miR-146a-5p, and miR-143-3p. Previous studies have revealed that miR-148a-3p [33, 34], miR-146a-5p [35–37], miR-24-3p [38, 39], and miR-30d-5p [40, 41] alleviate the I/R process by regulating inflammation, BBB damage, immune response, and neuronal injury. Notably, this study found that BMSC-exo effectively increased the levels of miR-148a-3p, miR-30d-5p, and miR-146a-5p in brain tissues of the MCAO-HT model, suggesting that they are one of the reasons why BMSC-exo ameliorated BBB injury and HT. This study focuses on miR-21a-5p because its differences in in the brain tissues of the groups were most pronounced. miR-21a-5p belongs to the miR-21 family and exerts functions such as neuroprotection and cardioprotection [42, 43], alveolar bone regeneration [44], inflammation inhibition [45], and immunomodulation [46]. In ischemic-hypoxic injury, miR-21a-5p delivered by extracellular vesicles of MSCs effectively alleviated brain injury by inducing microglia M2 polarization [47, 48]. Similarly, this study revealed that miR-21a-5p was low-expressed in the MCAO-HT animal model and the BBB injury model. Emphatically, miR-21a-5p overexpression enhanced the therapeutic effects of BMSC-exo on BBB injury and HT, suggesting a critical role for miR-21a-5p. Mechanistically, this study found that miR-21a-5p suppressed the activity of the TLR2/NF-κB pathway in vivo and in vitro. IS leads to necrosis or apoptosis of neurons, glial cells and endothelial cells, triggering damage-associated molecular patterns (DAMPs) [49–51]. TLR2, a receptor recognizing damage-associated molecular patterns (DAMPs), triggers a downstream signaling cascade culminating in the nuclear translocation of NF-κB. Activated NF-κB initiates the transcription of pro-inflammatory genes, triggers and amplifies the inflammatory cascade response, directly disrupts the integrity of the BBB, and creates pathological conditions for HT [49–51]. Therefore, miR-21a-5p-mediated inhibition of the TLR2/NF-κB pathway is responsible for the amelioration of BBB injury and HT by BMSC-exo.

Although this study systematically elucidated the role and mechanisms of BMSCs-exo and miR-21a-5p in ameliorating BBB injury and HT in IS, there are still some limitations that need to be considered. This study identifies that miR-21a-5p affects the levels and enzymatic activities of MMP-2 and MMP-9 and tight junction proteins via regulating the TLR2/NF-κB pathway, which provides important clues for understanding its role. However, key direct molecular targets have not been clearly validated. Moreover, this study focused on miR-21a-5p, but small RNA sequencing showed that BMSCs-exo contained 571 miRNAs and almost all the TOP10 miRNAs were differentially expressed in brain tissue. These miRNAs potentially regulate BBB injury and HT through synergistic or antagonistic effects, whereas functional validation of single miRNAs may underestimate the overall effect of exosomes. These are important directions for future research.

In summary, this study elucidated for the first time that BMSC-exo inhibits the TLR2/NF-κB pathway by delivering miR-21a-5p, thereby ameliorating BBB injury and HT after I/R. These finding offer a critical experimental foundation and guidance for developing novel therapeutics based on BMSCs-exo or its key active ingredient, miR-21a-5p, aimed at enhancing clinical outcomes in patients with IS, especially for the prevention and treatment of BBB injury and HT.

## Supplementary Information

Below is the link to the electronic supplementary material.

Supplementary file1 (PDF 8665 KB)

Supplementary file2 (XLSX 55 KB)

Supplementary file3 (XLSX 235 KB)

Supplementary file4 (XLSX 103 KB)

## Data Availability

The data are available on request from the corresponding author.
