# Supplementary figures and images for: BMSC-Derived Exosomal miR-21a-5p Ameliorates Blood–Brain Barrier Injury and Hemorrhagic Transformation
Source: Mol Neurobiol. 2026 Jan 8;63(1):351. doi: 10.1007/s12035-025-05650-6 (PMC12783216; doi:10.1007/s12035-025-05650-6)

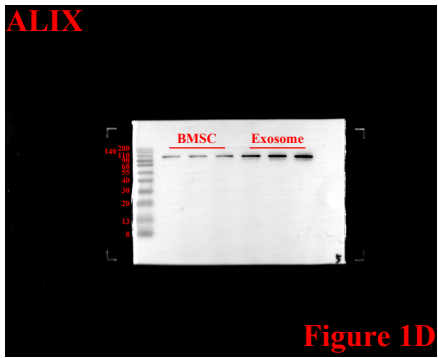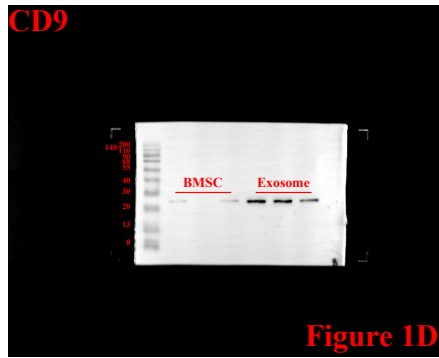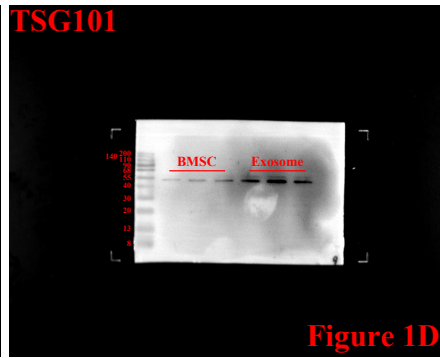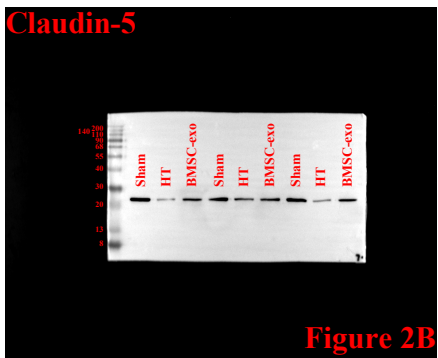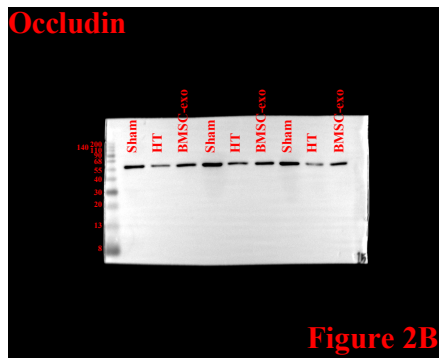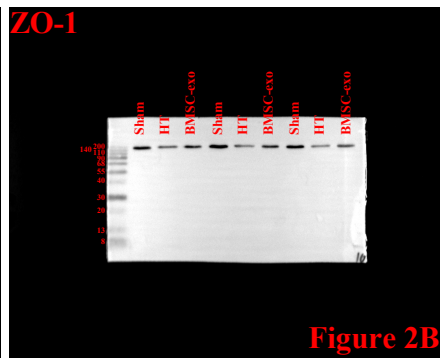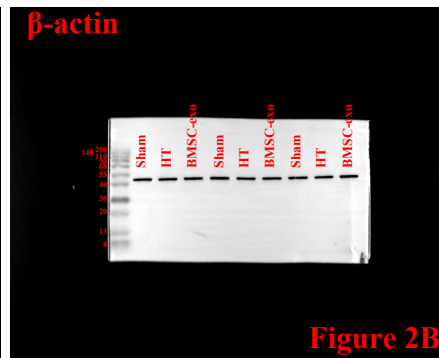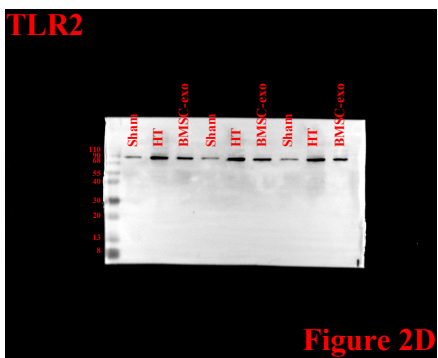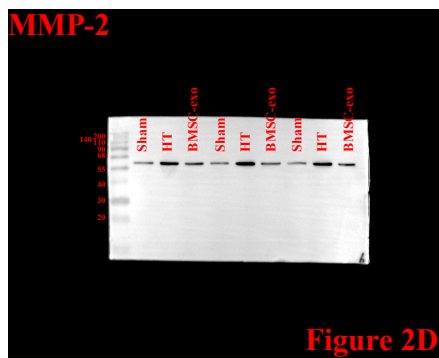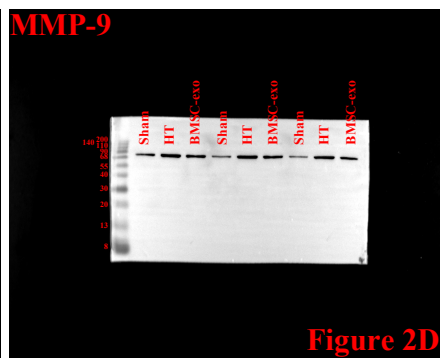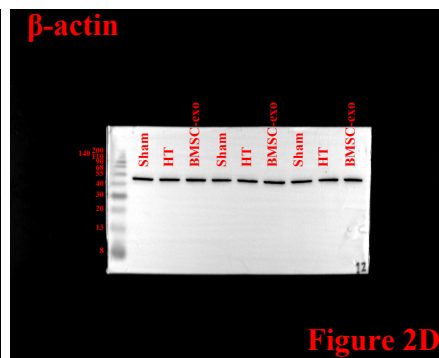

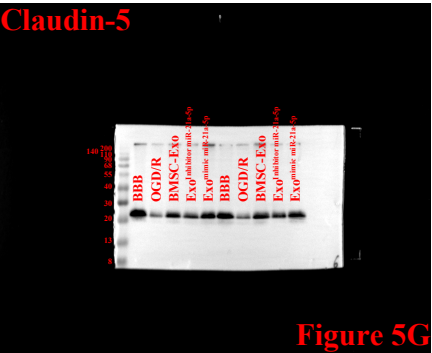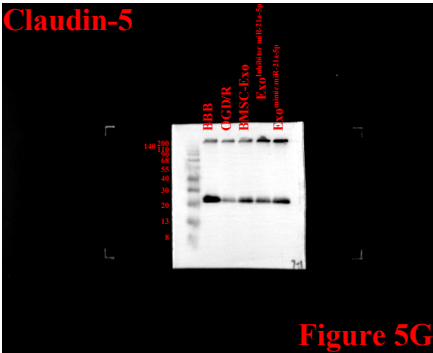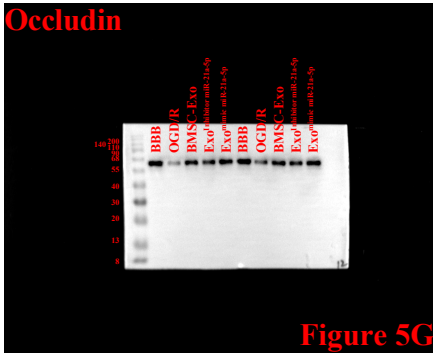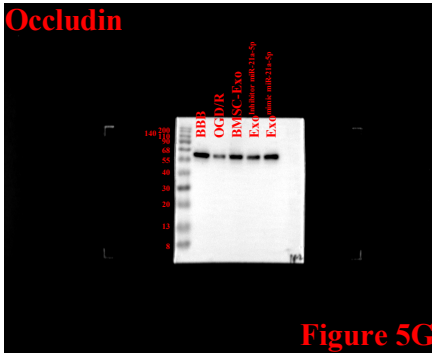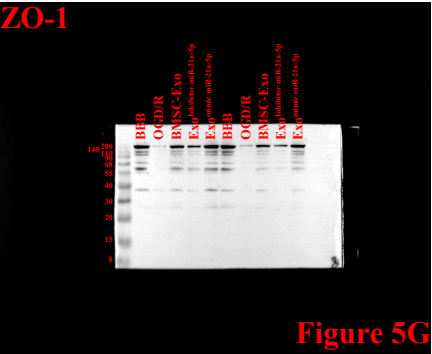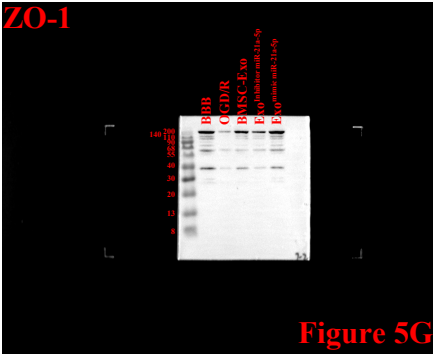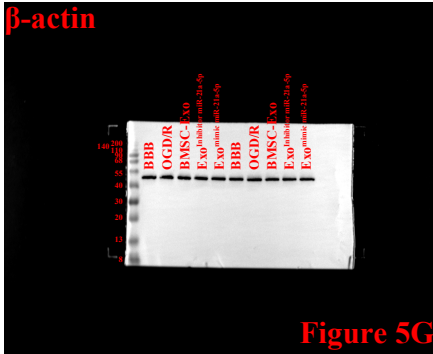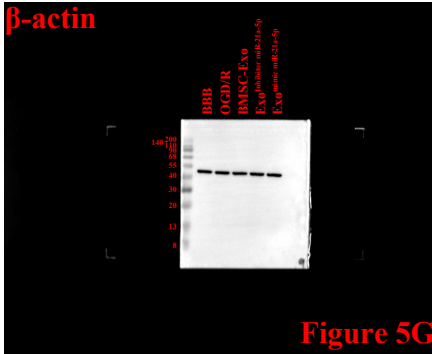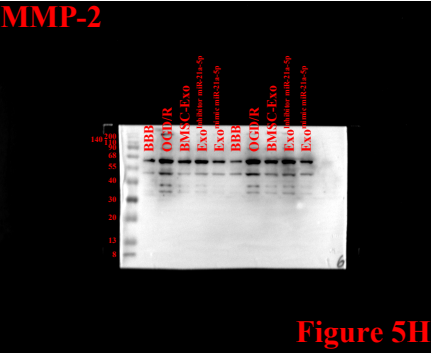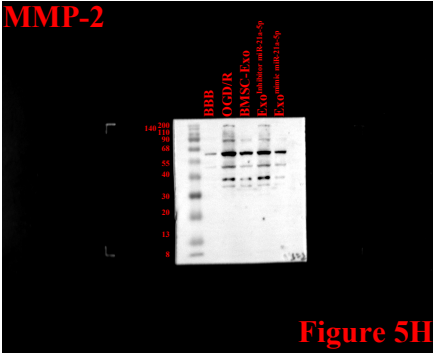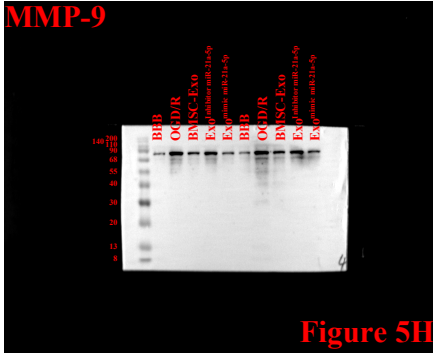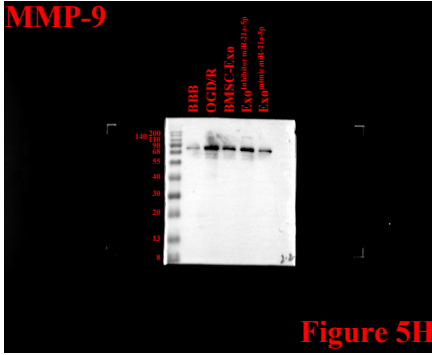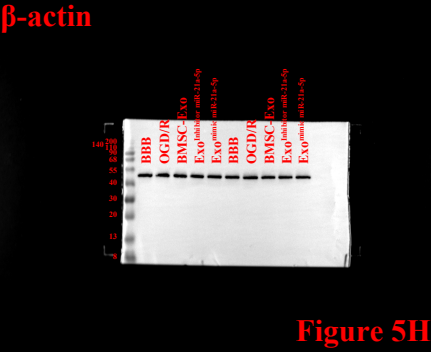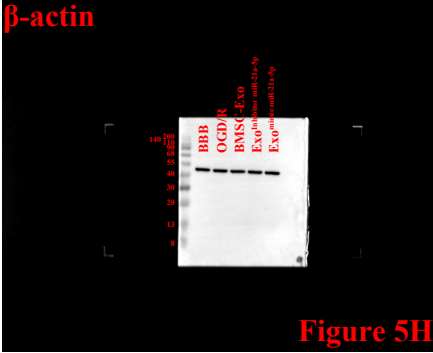

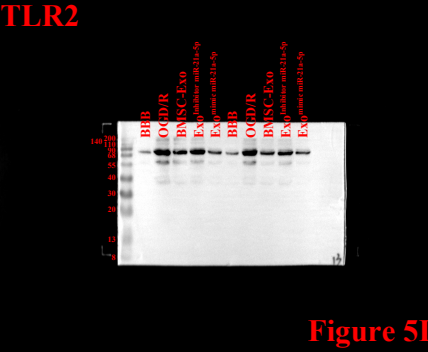

Figure 5I

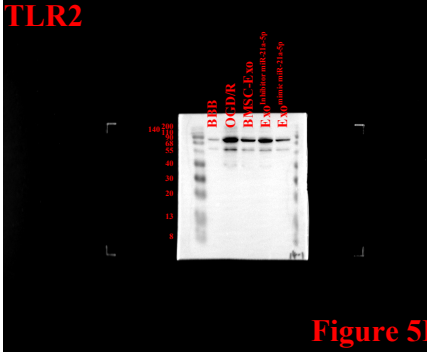

Figure 5I

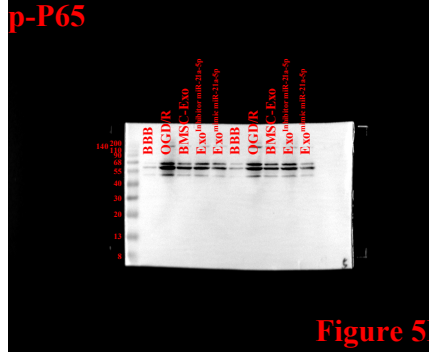

Figure 5I

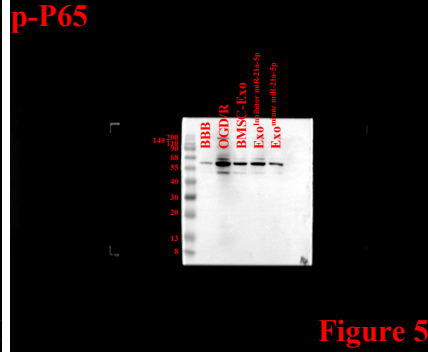

Figure 5I

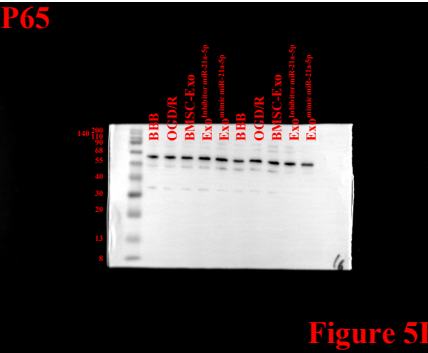

Figure 5I

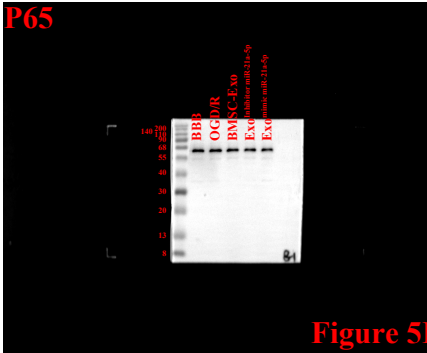

Figure 5I

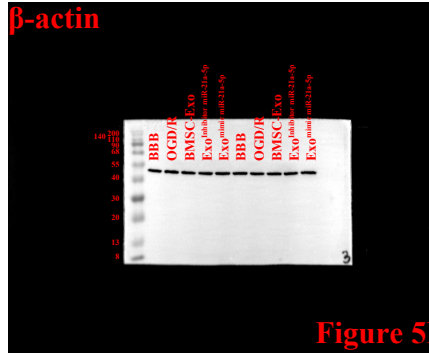

Figure 5I

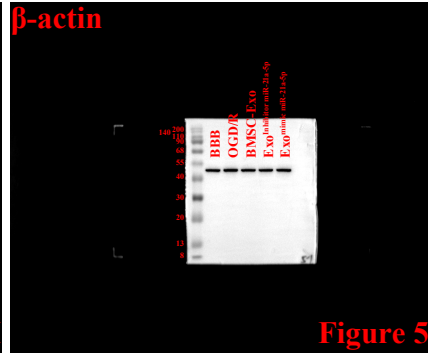

Figure 5I

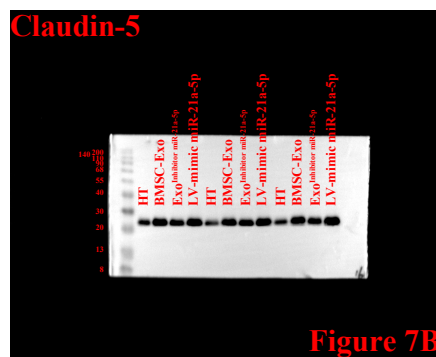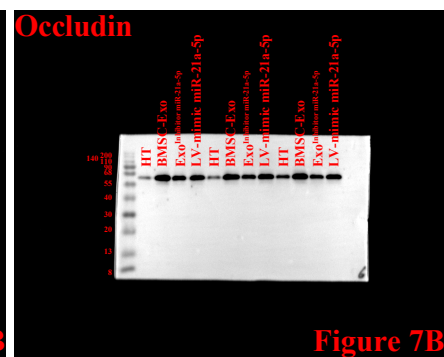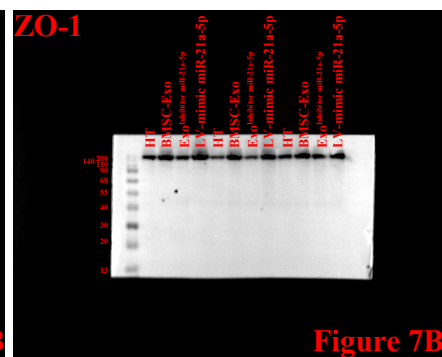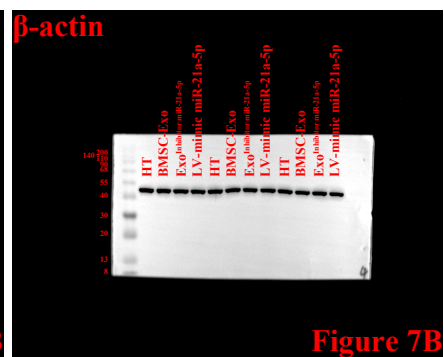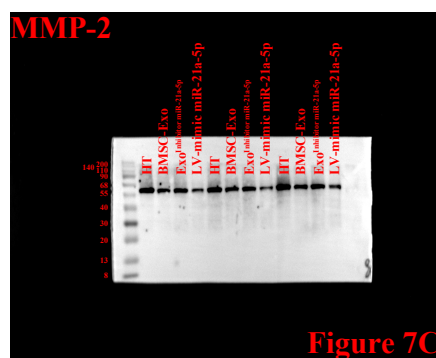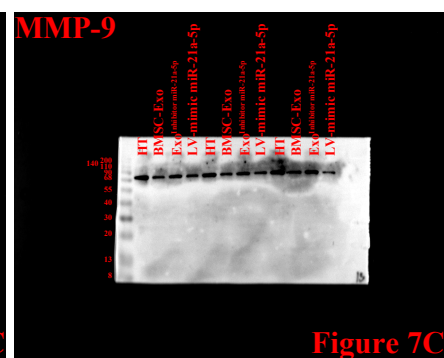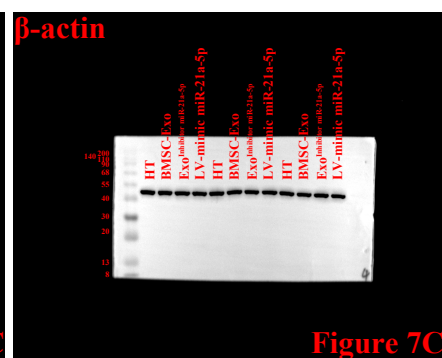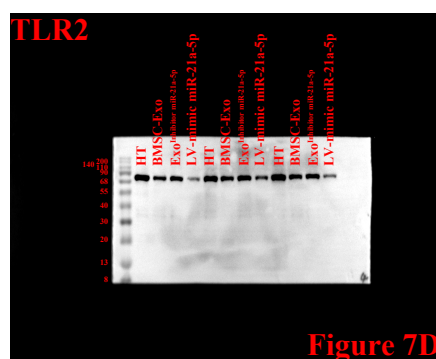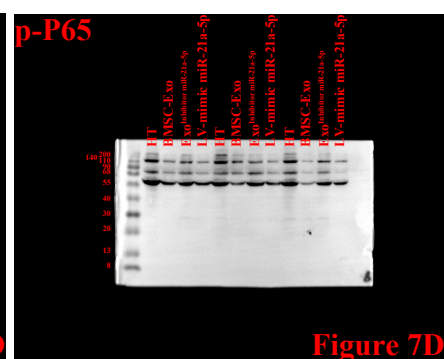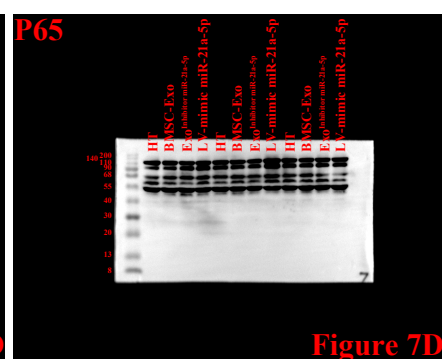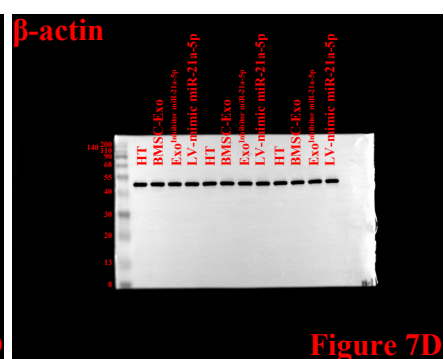

Supplement: Supplementary file 1 — Supplementary file1 (PDF 8665 KB) [file 12035_2025_5650_MOESM1_ESM.pdf]
